# Quality of Life in Hepatocellular Carcinoma Patients Treated with Transarterial Chemoembolization

**DOI:** 10.1155/2016/6120143

**Published:** 2016-04-07

**Authors:** Saleem Ahmed, Nurun Nisa de Souza, Wang Qiao, Meidai Kasai, Low Jee Keem, Vishal G. Shelat

**Affiliations:** ^1^Department of General Surgery, Tan Tock Seng Hospital, Singapore 308433; ^2^Ministry of Health Holdings, 1 Maritime Square, Singapore 099253; ^3^Duke-NUS Graduate Medical School, 8 College Road, Singapore 169857; ^4^Singapore Clinical Research Institute, 31 Biopolis Way, Singapore 138669; ^5^Department of Gastroenterological Surgery, Sendai Kousei Hospital, 8-15 Hirosemachi, Aoba-ku, Sendai-shi, Miyagi 9800873, Japan

## Abstract

Hepatocellular carcinoma (HCC) is one of the most commonly diagnosed cancers worldwide. Majority of patients with HCC are diagnosed in the advanced stages of disease and hence they are only suitable for palliative therapy. TACE (transarterial chemoembolization) is the most commonly used treatment for unresectable HCC. It is however unclear if TACE improves the quality of life (QoL) in patients with HCC. The aim of this review is to evaluate the impact of TACE on QoL of HCC patients.

## 1. Introduction

Hepatocellular carcinoma (HCC) is the 5th most common cancer worldwide and the 3rd most common cause of cancer-related death [1]. At diagnosis, fewer than 20% of patients are eligible for curative treatment [2]. The majority of patients receive palliation because of late-stage presentation, multiple comorbidities, associated hepatic dysfunction, and limited donor liver availability. The aim of palliative therapy is to provide symptomatic relief, extend survival, and improve QoL (quality of life).

Most advanced cancers are incurable and 95% of patients with advanced cancer report that QoL is at least as important as length of survival [3]. Palliative treatments may negatively influence QoL, especially if complications ensue. Poor QoL after treatment has a negative impact on the willingness of patients to continue and comply with future treatments. QoL is most influenced by health and healthcare interventions and hence QoL is an important clinical endpoint and it has become a component of clinical trials on chronic or incurable diseases [3].

TACE (transarterial chemoembolization) is the most widely used treatment for unresectable HCC [4] and is recommended as first-line treatment option for patients who meet the criteria for the intermediate stage of the Barcelona Clinic Liver Cancer (BCLC) staging system [5, 6]. Meta-analysis of six randomized controlled trials, including a total of 503 patients, showed survival benefit in patients who underwent TACE compared to the control group [7]. It is unclear if TACE helps in enhancing QoL of HCC patients by palliating several disturbing symptoms such as pruritis, fatigue, sleep disorders, sexual dysfunction, and abdominal discomfort [8]. Moreover, TACE can also cause postembolization syndrome, acute liver decompensation, or associated complications which can negatively affect the QoL. Hence it is important to study if HCC patients undergoing TACE enjoy a reasonably good QoL along with improved survival. The aim of this systematic review is to describe the current evidence and evaluate the impact of TACE on QoL of HCC patients.

## 2. Materials and Methods

### 2.1. Search Strategy

We searched medical databases including PubMed and SCOPUS for studies that discussed quality of life and/or survival rates of TACE. Search terms were (liver cancer OR hepatocellular carcinoma) AND (quality of life) AND (chemo^*∗*^ OR transarterial^*∗*^ OR infusional OR TACE) within the titles, abstracts, and keywords. In order to obtain a highly sensitive yield, we did not apply limits to our search. In addition, we hand searched the proceedings of conferences on liver diseases (International Hepato-Pancreato-Biliary Association) in 2011, 2012, and 2013.

All titles and abstracts of studies identified in the initial search were screened by lead author Vishal G. Shelat based on the following inclusion and exclusion criteria: (1) study population to consist of patients with hepatocellular carcinoma, (2) patients who were managed with transarterial chemoembolization, and (3) reported quality of life (QoL) outcomes using a discrete QoL tool. In studies reporting on outcomes for two or more groups including TACE as a control group, QoL outcomes of TACE group were included (2,9–12). Full-text papers of the selected studies were screened independently by Nurun Nisa de Souza and Vishal G. Shelat to assess eligibility. Any disagreements on eligibility were resolved by a third reviewer (Saleem Ahmed). Author Wang Qiao assisted in translation and analysis of the Chinese language study and Meidai Kasai assisted in translation and analysis of the Japanese language study.

We also included studies reporting on infusional chemotherapy without embolization [9, 10]. Exclusion criteria ruled out any study reporting on QoL in HCC patients treated by radiofrequency ablation, radioembolization, hepatic resection, or liver transplantation only. References of all the included studies were screened for potentially relevant studies not identified during initial search.

### 2.2. Data Extraction

The following variables were extracted from the studies where available: number of patients, age, sex, QoL questionnaire used, timing of the questionnaire administration, and dosage and type of chemotherapy agent used in TACE and QoL outcomes.

## 3. Results

**Figure 1:**
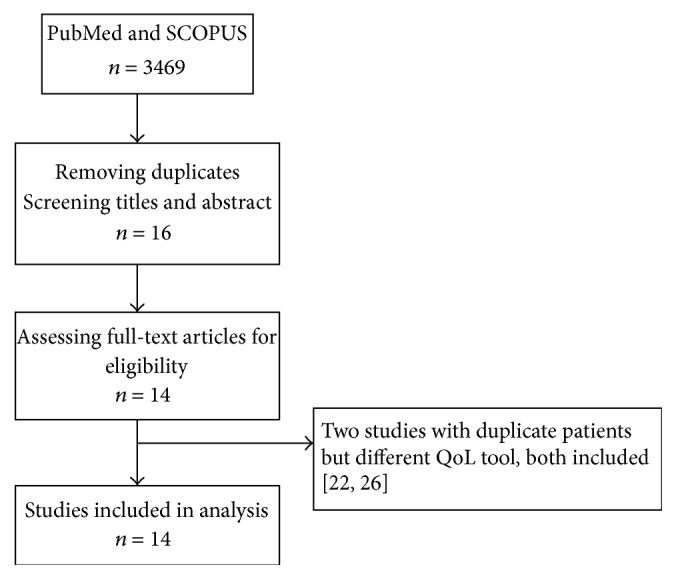
Study selection.

We identified a total of 3469 studies (Figure 1) through electronic searches and from the reference lists of eligible articles. Of these, 3453 were excluded after reading titles and abstracts and further two studies were excluded for reporting QoL in patients who did not undergo TACE.

The description of the QoL tools used in the 14 publications studying the impact of TACE on QoL is detailed in Table 1. All studies except one (Shun et al.) used a single QoL instrument to assess impact of QoL in patients who underwent TACE [22]. Four authors used their own unique questionnaire [9, 10, 19, 20].

### 3.1. QoL Tools

#### 3.1.1. Review of Studies Reporting on QoL in TACE Patients

Based upon the literature search, 14 studies were identified which reported on the use of QoL instruments to study the impact of TACE on QoL of HCC patients.  Table 2 provides a summary of the studies, the sample size, comparative groups if any, disease profile, and the details of the TACE regime used in the study.

All studies that had one or more comparative groups compared the QoL of patients undergoing TACE alone versus other treatment strategies except the study by Wang et al. who compared TACE alone versus TACE and RFA in combination [23].

The study by Toyoda et al. studied the effect of repetitive continuous local intra-arterial injection chemotherapy with 5-FU and CDDP (5-FU 50 mg/day + CDDP 5–10 mg/day) via implantable reservoir on QoL. Owing to the small numbers for QoL analysis it did not produce statistically significant results. However, it found that among the 3 patients with partial remission 2 of them had an improvement in QoL scores. The overall at-home rate was 94% and the main reason for admission was to troubleshoot catheters.

Shun et al.'s study in 2012 shows that all 8 domains of QoL scores (short form 12 item health survey) improved over the three time periods studied (prior to discharge and at 4th and 8th weeks after discharge) except for vitality which improved only after 2 months after discharge. The study by Jianbo et al. showed that the QoL improved at 1 and 3 months in the physiology and symptom domains of the questionnaire compared with preintervention [20]. However, the psychology domains fell back to preintervention levels and the social domain was worse as compared to preintervention levels. Shun et al.'s study in 2005 supplements the data by Jianbo et al. on symptoms and fatigue by describing QoL scores in the more immediate period after intervention (up to day 6 after intervention) [20, 22]. It shows that the scores for fatigue, symptom distress, and depression peaked (worse) on day 2 and subsequently trended lower on day 6, although they were still higher than preintervention levels. Wible et al.'s study shows that at the 4th month after intervention there was a significant improvement in mental health scores in contrast to the data by Jianbo et al., which show that the psychology domain scores fell back to preintervention levels at the third month [20, 24]. The study by Wible et al. also showed that there was no trend towards deterioration of patient's overall QoL over a 1-year period.

Eltawil et al.'s prospective observational study designed to assess both survival and QoL of primary HCC patients showed that there was a stable trend of QoL of patients undergoing repeat sessions of TACE over time [27]. The study did not see any statistically significant temporal trends for any of the four QoL domains, although there was tendency towards declining physical health after the 3rd session of TACE which is approximately 1 year after 1st session. This finding is supported by Wible et al. and Xing et al., both showing that the patients are able to tolerate repeated TACE over a period of 1 year with no significant drop in QoL. However, Toro et al. compare QoL of patients who undergo different intervention and measure QoL over a 24-month period. Patients who underwent TACE show statistically significantly worse QoL in all domains at the 12th month and 24th month marks compared to the 3rd month in Toro et al.'s study [25]. Xie et al. have multiple time points for conducting the QoL questionnaire from the 1st month after procedure all the way to 24 months [28]. Generally, there is sharp decline in the physical and mental components in the 1st month; however, both recover in the 3rd month and 6th month and start to decline again in the 12th month mark with the worst scores being recorded at the 24th month mark.

The study by Kato et al. is the earliest study in our review to study effect of TACE on QoL. They use their own unique tool to study QoL. It is a 10-item questionnaire with a 5-point ordinal scale. There is no overall QoL score reported and each item in the questionnaire is compared individually. The study is one of 2 studies that study at-home rates as a surrogate measure of QoL. The results of the study by Kato et al. suggest that anorexia and depression symptoms were particularly worse in patients who underwent TACE with doxorubicin, while those who underwent TAE had much worse abdominal pain, fatigue, and uneasiness scores. Those patients who underwent MMC microcapsule therapy had the highest at-home rates of 86.6%, while those who underwent TACE with doxorubicin had the lowest at-home rates of 43.5%.

The studies included in this review tended to be with high risk of bias [30].

## 4. Discussion

QoL is both clinically and physiologically meaningful endpoint and is best defined from the patient's perspective [31]. Ferrel defines QoL in cancer as a personal sense of well-being encompassing a multidimensional perspective that generally includes physical, psychological, social, and spiritual dimensions or domains [32–35]. Changes in one domain can affect or influence other domains.

**Figure 2:**
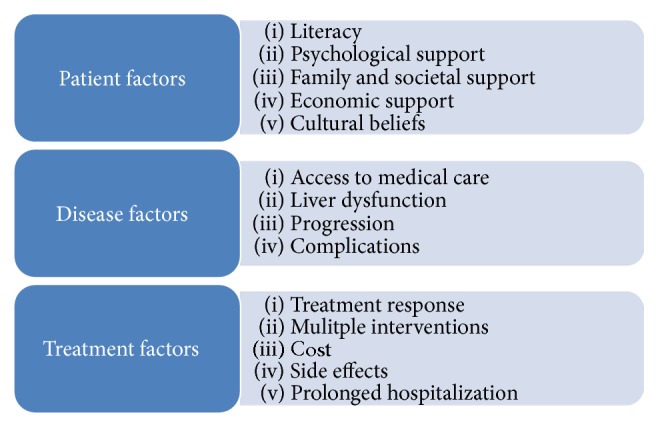
Factors influencing QoL in HCC patients treated with TACE.

HCC is a common cancer and patients often present at late stages. TACE is the most common palliative treatment modality and recent meta-analysis has demonstrated survival benefit of TACE. There are limited data to show the effect of TACE on QoL [8]. It is important that current evidence on this topic is summarized and synthesised. Due to heterogeneity in existing reports, a meta-analysis is not possible. There is variation in selection of HCC patients, the TACE treatment protocols, and QoL measures employed in the 14 studies, which all contribute to heterogeneity and make direct comparison difficult.  Figure 2 provides a summary of patient, disease, and treatment factors affecting QoL in HCC patients treated with TACE.

### 4.1. Study Populations

Eltawil et al.'s study included HCC patients with disease not amenable to ablation or resection, while the study by Toro et al. included patients with primary HCC who were eligible for resection, ablation, and TACE [25, 27]. Furthermore, the study by Toyoda et al. included only patients with stage IVa HCC, while the study by Tanabe et al. only looked at patients with HCC recurrence after initial curative resection [9, 10].

### 4.2. Treatment

The treatment protocols for TACE vary from institution to institution and also vary according to the time period studied. There is no strong evidence to favour one chemotherapy agent over another agent. This gives rise to variance in dose, concentration, rate of injection of drug, and even the choice of embolizing agent or its volume to be used if it is at all used [36]. There is even variance in the number of chemotherapy agents used: some authors such as Tanabe et al. use single agent chemotherapy, while others such as Jianbo et al. use combination of up to 3 chemotherapy agents [10, 20]. Intuitively, use of combination chemotherapy may produce synergistic effects with less toxicity due to lower dose of individual chemotherapeutic agents; however, there are few data to support this [37]. Toyoda et al.'s study was the only study to use continuous infusion of chemotherapy.

### 4.3. QoL Questionnaires

The choice of QoL questionnaires is also variable with 4 of the 14 studies using their own unique questionnaires and, even among those using standardized questionnaires, the number of items and content of the scales vary between instruments making direct comparison difficult [38]. Furthermore, the timing of administration of questionnaires and the chemotherapy agent used also may influence QoL. All this is compounded by the fact that some of the studies are statistically underpowered to provide conclusive results.  Figure 2 demonstrates all the potential factors which can influence QoL of HCC patients treated with TACE. There is no study which is ideal and, therefore, we report a descriptive systematic review to evaluate impact of TACE on QoL of HCC patients.

### 4.4. QoL Assessment

QoL is subjective and multifaceted [8]. The ability to understand QoL is only as good as the tools available. QoL questionnaires are essential tools in quantifying the physical, social, psychological, and spiritual domains and can generally be categorised as generic, disease-specific, and symptom-specific. The generic instruments measure the complete range of diseases in different populations and are particularly useful in comparing QoL across different diseases [39]. The disease-specific instruments measure domains of QoL specific to a disease process. Carolinas Comfort Scale for hernias is an example of such a scale [40]. Symptom-specific instruments measure QoL changes specific to a symptom, for example, nausea [39].

A recent review of an online database of QoL tools (http://proqolid.org/) produced over 50 neoplasia-specific and 2 hepatobiliary-specific tools [41]. For a QoL instrument to be useful it must be able to satisfy the basic psychometric principles of validity, reliability, and responsiveness in the patient population studied [8, 42]. Clinical utility, ease of administration, and scoring are other important factors impacting the usefulness of the HRQoL instrument [42].

Despite the large number of neoplasia and hepatobiliary-specific QoL tools available, 4 out of 14 (29%) studies still used their own unique questionnaire [9, 10, 19, 20]. The high number of unique questionnaires could be due to 2 main reasons. Firstly, of the 4 studies that used their own unique questionnaires, 3 were non-English publications (2 Japanese and 1 Chinese). Lack of ready translated HRQoL questionnaires might have prompted the authors to use their own unique questionnaires. Moreover, since most HRQoL tools are developed in the West, they might have not undergone validation in the Asian setting, as QoL is subjective in nature and is influenced by the cultural and social norms of the population studied. Secondly, 2 of the papers (Kato et al. and Toyoda et al.) were published in the 1990s when the tools for HRQoL have not yet gained widespread prominence and most of the tools available then were generic in nature. Generic QoL instruments lack detail to assess the impact of symptoms specific to the disease state.

However, these issues are being addressed with the development of many HRQoL tools specifically aimed at patients with HCC. For example, QLQ-HCC18 of European Organisation for Research and Treatment of Cancer (EORTC) has been developed for use specifically in patients with HCC as a supplement module to EORTC QLQ-C30 [43]. It is the first questionnaire to include patients from both East and West during its development and included patients from Europe, Taiwan, and Hong Kong. To add further credibility, in addition to literature search, the questionnaire was developed using semistructured interviews with patients and healthcare professionals. In addition, it is currently available in Arabic, English, Chinese, and Taiwanese and is in the process of being validated. It is likely that more authors would be using internationally validated HRQoL tools with ready translations available in their own local languages. This would enable more meaningful comparison of study data across different languages and cultural settings.

Functional Assessment of Cancer Therapy (FACT) QoL tool is the most commonly used tool among the publications included in this review. FACT-G is a neoplasia-specific HRQoL tool developed in 1993 [12] and is one of the most widely used QoL instruments for cancer patients [8]. FACT-G was developed by answers generated from open-ended interviews with patients and oncology professionals. The 28-item questionnaire, in addition to a total score, also produces subscale scores for physical, social, and emotional well-being as well as satisfaction with treatment relationship. FACT-Hep is a hepatobiliary neoplasia-specific tool adapted from the Functional Assessment of Chronic Illness Therapy (FACIT) measurement system [3]. In addition to the questions contained in the original FACT-G scales, there are additional 18 questions to assess symptoms and QoL issues specific to patients with hepatobiliary cancers. The items are scored from 0 to 4, with higher overall and subscale scores pointing to better QoL. It is known to have good test-retest reliability. FACT-G and FACT-Hep together were used in 3 of the 12 studies.

Despite the difference in study variables, there are some general outcomes that are observed in the 14 studies. Firstly, as shown in the studies by Tanabe et al. and Toro et al., resection of tumour with a curative intent provides better overall QoL than TACE [10, 25]. While Toro et al. suggest that the overall QoL is worse in patients that underwent RFA compared to TACE, the combination of RFA and TACE was shown to be superior in terms of QoL outcomes compared to TACE alone in the study by Wang et al. [23, 25]. However, when compared with ^90^Yttrium radioembolization, TACE offers inferior QoL outcomes [2, 21].

QoL is dynamic measurement encompassing many dimensions which change independently of each other over time. The timing of the administration of the QoL tool to the patients is important and can influence study outcomes. The time of administration of questionnaire after intervention can be divided into early (≤1 month after intervention), intermediate (1–3 months after intervention), and late (≥3 months after intervention). Administration of the questionnaire in the early stage may negatively influence scores for symptom and physical and psychological domains. This is because patients might experience pain from procedure and this coupled with complications can lead to physical, functional, and psychological distress. Shun et al. noted that scores for symptom distress, fatigue, anxiety, and depression peaked on 2nd day after intervention and subsequently trended lower on the 6th day after intervention [22]. Administration of questionnaire in the intermediate phase can have varying results depending on treatment response and patients' knowledge of treatment effectiveness. The studies that looked at the intermediate term such as the studies by Jianbo et al. and Wible et al. generally showed improved QoL [20, 24]. Administration of questionnaire in long term can be affected by many aspects including but not limited to disease progression and economic aspects. The studies that reported on long-term QoL showed differing results. The studies by Wible et al. and Xing et al. show no significant drop in QoL at 1 year, while the studies by Toro et al. and Xie et al. show statistically significantly worse QoL at the 1-year and 2-year time periods [24, 25, 29]. It is likely that psychological domain will be negatively influenced in patients who know that they have disease progression.

However, there is limited evidence to suggest that clinicians actually study the QoL in HCC patients undergoing palliative TACE. The existing studies are heterogeneous with regard to the type of QoL tool used and timing of administration of the questionnaire. This is further compounded with geopolitical, socioeconomic, and cultural values of global population.

## 5. Conclusion

QoL measurement has become an important outcome measurement in oncology, especially in the palliative setting. However, there is limited robust evidence to conclusively derive the impact of TACE on QoL in patients with HCC. It is important that hepatobiliary oncology community recognize measurement of QoL as an important aspect of multidisciplinary patient care and international collaboration is sought to standardize the measurement of QoL in HCC patients treated with palliative TACE.

## Figures and Tables

**Table 1:** Description of quality of life instruments used in HCC patients undergoing TACE.

Instruments	Domains (items)	Domain description
FACT-Hep QoL Questionnaire [11]	5 (45)	Emotional well-being, functional well-being, and physical well-being Social/family well-being Additional concerns

FACT-G QoL Questionnaire [12]	4 (28)	Emotional well-being, functional well-being, physical well-being, and social/family well-being

WHOQoL-BREF Questionnaire [13]	4 (26)	Physical health Psychological health Social relationships Environment

SF-36 [14]	8 (36)	Physical functioning Role physical Bodily pain General health Vitality Social functioning Role emotional Mental health

SF-12 [15]	8 (12)	Same as SF-36

Revised Piper Fatigue Scale (PFS) [16]	4 (27)	Behavioural/severity, affective meaning, sensory, and cognitive/mood

Modified Symptom Distress Scale (SDS-m) [17]	1 (13)	Symptoms: nausea (frequency, intensity), appetite, insomnia, pain (frequency, intensity), fatigue, bowel patterns, concentration, appearance, breathing, outlook, and cough

Hospital Anxiety and Depression Scale (HADS) [18]	2 (14)	Anxiety and depression

Kato et al. [19]	3 (10)	Physical health Social well-being Additional concerns regarding confidence in treatment

Tanabe et al. [10]	4 (4)	Physical function, psychological function, social function, and physical sensation

Toyoda et al. [9]	2 (10)	Symptoms Psychology Social function

Jianbo et al. [20]	4 (22)	Physiology Psychology Symptoms Social function

FACT: Functional Assessment of Cancer Therapy; WHO: World Health Organization; QoL: Quality of Life; SF: short form.

**Table 2:** Impact of TACE on QoL in patients with HCC.

SN	Author	Study design and population	Intervention	Comparative groups or subset	Timing of questionnaire	Outcome
1	Kato et al. 1990 [19]	Prospective study Unresectable HCC *n* = 16	Patients with unresectable liver cancer: comparison of TAE, MMC microcapsule, and single shot intra-arterial doxorubicin	6: TAE; 6: MMC microcapsule; 4: single shot intra-arterial doxorubicin	Before TACE 1 week after 2 weeks after	Anorexia and depression worse in patients who underwent TACE with doxorubicin

2	Toyoda et al. 1993 [9]	Retrospective study Stage IVa HCC *n* = 21	Patients with stage IVa HCC who are unsuitable for surgery, PEIT, and TAE were selected for continuous intra-arterial chemotherapy	NA	Variable timing	Two patients in partial remission group had improvement in QoL

3	Tanabe et al. 2001 [10]	Retrospective study HCC recurrence after initial curative resection *n* = 23	Recurrence of HCC after HR: comparison of repeat HR versus HAI chemotherapy	13: HAI; 10: repeat HR	After TACE	Repeat HR provides good prognosis and favourable QoL compared to HAI in patients with resectable recurrence

4	Jianbo et al. 2002 [20]	Prospective study Primary HCC *n* = 175	TACE	NA	Before TACE 1 month after 3 months after	QoL is improved after TACE and can be maintained till 3 months after treatment in the physiology, psychology, and symptoms domain

5	Steel et al. 2004 [21]	Prospective nonrandomized cohort study Histology proven HCC *n* = 28	Patients with HCC: comparison of TACE and ^90^Y radioembolization	14: TACE; 14: ^90^Y radioembolization	Before TACE 3 months after 6 months after 12 months after	Treatment with Yttrium has a modest advantage with regard to QoL when compared to HAI with cisplatin

6	Shun et al. 2005 [22]	Prospective study Primary HCC *n* = 40	TACE 16 patients had 2–5 previous TACE procedures	NA	Before TACE 2 days after 4 days after 6 days after	Patient fatigue levels peaked at day 2. Factors responsible for increased fatigue levels include greater symptom distress, anxiety, and depression, higher Adriamycin dosage, longer duration of previous fatigue; and less education levels

7	Wang et al. 2007 [23]	Prospective randomized study Histology proven HCC *n* = 83	Patients with HCC: comparison of TACE and TACE + RFA	40: TACE; 43: TACE + RFA	Before TACE 3 months after 1st TACE	The overall QoL of HCC patients in TACE-RFA group was maintained at higher level than that of TACE group

8	Wible et al. 2010 [24]	Prospective study Primary HCC *n* = 73	TACE 23 patients underwent 3 or more TACE procedures	NA	Before TACE 4 months after 8 months after 12 months after	Patients with HCC are likely to perceive improved mental health during the first 4 months of primary TACE. If they undergo more than 2 procedures, they are likely to perceive improved mental health during the first 2 sessions. Patient-perceived vitality will likely worsen after initial procedure

9	Toro et al. 2012 [25]	Prospective study Primary HCC eligible for HR, RFA, TACE, or NT *n* = 52	Patients with HCC: comparison of HR, TACE, RFA, and NT	14: HR; 15: TACE; 9: RFA; 13: NT	Before TACE 3 months after 6 months after 12 months after 24 months after	RFA provides a worse QoL compared to HR but a higher QoL compared to TACE or NT

10	Shun et al. 2012 [26]	Prospective study Primary HCC patients receiving TACE *n* = 89	TACE	NA	3 days prior to discharge 4 weeks after discharge 8 weeks after discharge	Those at greatest risk for lower QoL include males and those who have higher levels of depression and anxiety after discharge

11	Eltawil et al. 2012 [27]	Prospective study Primary HCC not amenable to ablation or resection *n* = 48	TACE 6 patients underwent 3 or more TACE procedures	NA	Data collected every 3-4 months	QoL remained stable for almost a year and only started to decline after the 3rd TACE which coincided with progression of tumour; concluded that majority of patients were able to tolerate several TACE sessions without significant deterioration of QoL

12	Salem et al. 2013 [2]	Prospective study Primary HCC *n* = 56	Patients with HCC: comparison of TACE versus ^90^Y radioembolization	29: ^90^Y radioembolization; 27: TACE	Before TACE 2 weeks after 4 weeks after	QoL difference did not reach statistical significance; change in EES score was most pronounced; ^90^Y radioembolization is better able to maintain health-related QoL.

13	Xie et al. 2015 [28]	Retrospective study Primary intermediate stage HCC *n* = 102	Patients with HCC: comparison of TACE versus HR	58: HR 44: TACE	Before TACE 1 month after 3 months after 6 months after 12 months after 24 months after	QoL was lower in the 1st month after procedure but recovered in the 3rd and 6th months but dropped again in the 12th month with lowest scores in the 24th month

14	Xing et al. 2015 [29]	Prospective study Unresectable HCC *n* = 118	TACE	NA	Before TACE 3 months after 6 months after 12 months after	QoL was preserved for up to 12 months after TACE

5-FU: 5-Fluorouracil; CDDP: cisplatin; HAI: hepatic artery infusion; HR: hepatic resection; MMC: mitomycin C; NA: not applicable; NS: not specified; NT: no treatment; QoL: quality of life; RFA: radiofrequency ablation; SD: standard deviation; TACE: transarterial chemoembolization; TAE: transarterial embolization.

## References

[1] Bosch F. X., Ribes J., Cléries R., Díaz M. (2005). Epidemiology of hepatocellular carcinoma. *Clinics in Liver Disease*.

[2] Salem R., Gilbertsen M., Butt Z. (2013). Increased quality of life among hepatocellular carcinoma patients treated with radioembolization, compared with chemoembolization. *Clinical Gastroenterology and Hepatology*.

[3] Heffernan N., Cella D., Webster K. (2002). Measuring health-related quality of life in patients with hepatobiliary cancers: the functional assessment of cancer therapy-hepatobiliary questionnaire. *Journal of Clinical Oncology*.

[4] Takayasu K., Arii S., Ikai I. (2006). Prospective cohort study of transarterial chemoembolization for unresectable hepatocellular carcinoma in 8510 patients. *Gastroenterology*.

[5] European Association for the Study of the Liver (2012). EASL-EORTC clinical practice guidelines: management of hepatocellular carcinoma. *Journal of Hepatology*.

[6] Bruix J., Sherman M. (2011). Management of hepatocellular carcinoma: an update. *Hepatology*.

[7] Llovet J. M., Bruix J. (2003). Systematic review of randomized trials for unresectable hepatocellular carcinoma: chemoembolization improves survival. *Hepatology*.

[8] Gandhi S., Khubchandani S., Iyer R. (2014). Quality of life and hepatocellular carcinoma. *Journal of Gastrointestinal Oncology*.

[9] Toyoda H., Nakano S., Takeda I. (1993). The study of continuous local arterial-infusion chemotherapy with 5-FU + CDDP for patients with severely advanced HCC—for the elongation of the life-span and the improvement of QOL. *Gan To Kagaku Ryoho*.

[10] Tanabe G., Ueno S., Maemura M. (2001). Favorable quality of life after repeat hepatic resection for recurrent hepatocellular carcinoma. *Hepato-Gastroenterology*.

[11] Heffernan N., Cella D., Webster K. (2002). Measuring health-related quality of life in patients with hepatobiliary cancers: the functional assessment of Cancer Therapy-Hepatobiliary Questionnaire. *Journal of Clinical Oncology*.

[12] Cella D. F., Tulsky D. S., Gray G. (1993). The functional assessment of cancer therapy scale: development and validation of the general measure. *Journal of Clinical Oncology*.

[13] (1998). Development of the World Health Organization WHOQOL-BREF quality of life assessment. The WHOQOL Group. *Psychological Medicine*.

[14] Ware J. E., Snow K. K., Kosinski M., Gandek B. (1993). *SF-36 Health Survey*.

[15] Ware J. E., Kosinski M., Keller S. D. (1996). A 12-item short-form health survey: construction of scales and preliminary tests of reliability and validity. *Medical Care*.

[16] Piper B. F., Dibble S. L., Dodd M. J., Weiss M. C., Slaughter R. E., Paul S. M. (1998). The revised Piper Fatigue Scale: psychometric evaluation in women with breast cancer. *Oncology Nursing Forum*.

[17] Holmes S. (1989). Use of a modified symptom distress scale in assessment of the cancer patient. *International Journal of Nursing Studies*.

[18] Zigmond A. S., Snaith R. P. (1983). The hospital anxiety and depression scale. *Acta Psychiatrica Scandinavica*.

[19] Kato T., Niwa M., Saito Y. (1990). Evaluation of quality of life in arterial infusion chemotherapy of hepatocellular carcinoma. *Gan To Kagaku Ryoho*.

[20] Jianbo Z., Yanhao L., Yong C. (2002). Evaluation of quality of life before and after interventional therapy in patients with primary hepatocellular carcinoma. *Chinese Journal of Radiology*.

[21] Steel J., Baum A., Carr B. (2004). Quality of life in patients diagnosed with primary hepatocellular carcinoma: hepatic arterial infusion of cisplatin versus 90-Yttrium microspheres (Therasphere®). *Psycho-Oncology*.

[22] Shun S.-C., Lai Y.-H., Jing T.-T. (2005). Fatigue patterns and correlates in male liver cancer patients receiving transcatheter hepatic arterial chemoembolization. *Supportive Care in Cancer*.

[23] Wang Y.-B., Chen M.-H., Yan K., Yang W., Dai Y., Yin S.-S. (2007). Quality of life after radiofrequency ablation combined with transcatheter arterial chemoembolization for hepatocellular carcinoma: comparison with transcatheter arterial chemoembolization alone. *Quality of Life Research*.

[24] Wible B. C., Rilling W. S., Drescher P. (2010). Longitudinal quality of life assessment of patients with hepatocellular carcinoma after primary transarterial chemoembolization. *Journal of Vascular and Interventional Radiology*.

[25] Toro A., Pulvirenti E., Palermo F., Di Carlo I. (2012). Health-related quality of life in patients with hepatocellular carcinoma after hepatic resection, transcatheter arterial chemoembolization, radiofrequency ablation or no treatment. *Surgical Oncology*.

[26] Shun S.-C., Chen C.-H., Sheu J.-C., Liang J.-D., Yang J.-C., Lai Y.-H. (2012). Quality of life and its associated factors in patients with hepatocellular carcinoma receiving one course of transarterial chemoembolization treatment: a longitudinal study. *Oncologist*.

[27] Eltawil K. M., Berry R., Abdolell M., Molinari M. (2012). Quality of life and survival analysis of patients undergoing transarterial chemoembolization for primary hepatic malignancies: a prospective cohort study. *HPB*.

[28] Xie Z. R., Luo Y. L., Xiao F. M., Liu Q., Ma Y. (2015). Health-related quality of life of patients with intermediate hepatocellular carcinoma after liver resection or transcatheter arterial chemoembolization. *Asian Pacific Journal of Cancer Prevention*.

[29] Xing M., Webber G., Prajapati H. J. (2015). Preservation of quality of life with doxorubicin drug-eluting bead transarterial chemoembolization for unresectable hepatocellular carcinoma: longitudinal prospective study. *Journal of Gastroenterology and Hepatology*.

[30] Hopp L. (21). Risk of bias reporting in Cochrane systematic reviews. *International Journal of Nursing Practice*.

[31] Gill T. M., Feinstein A. R. (1994). A critical appraisal of the quality of quality-of-life measurements. *The Journal of the American Medical Association*.

[32] Ferrell B., Grant M., Padilla G., Vemuri S., Rhiner M. (1991). The experience of pain and perceptions of quality of life: validation of a conceptual model. *Hospice Journal*.

[33] Ferrell B. R., Wisdom C., Wenzl C. (1989). Quality of life as an outcome variable in the management of cancer pain. *Cancer*.

[34] Ferrell B. R. (1995). The impact of pain on quality of life. A decade of research. *The Nursing Clinics of North America*.

[35] Ferrell B. R., Dow K. H., Grant M. (1995). Measurement of the quality of life in cancer survivors. *Quality of Life Research*.

[36] Guan Y.-S., He Q., Wang M.-Q. (2012). Transcatheter arterial chemoembolization: history for more than 30 years. *ISRN Gastroenterology*.

[37] Shin S. W. (2009). The current practice of transarterial chemoembolization for the treatment of hepatocellular carcinoma. *Korean Journal of Radiology*.

[38] Kaasa S., Loge J. H. (2002). Quality-of-life assessment in palliative care. *The Lancet Oncology*.

[39] MacKeigan L. D., Pathak D. S. (1992). Overview of health-related quality-of-life measures. *American Journal of Hospital Pharmacy*.

[40] Yeo A. E. T., Berney C. R. (2012). Carolinas comfort scale for mesh repair of inguinal hernia. *ANZ Journal of Surgery*.

[41] Mapi Research Trust PROQOLID, the Clinical Outcome Assessment (COA) Instruments Database. http://www.proqolid.org/.

[42] Slevin M. L. (1992). Quality of life: philosophical question or clinical reality?. *British Medical Journal*.

[43] Blazeby J. M., Currie E., Zee B. C. Y., Chie W.-C., Poon R. T., Garden O. J. (2004). Development of a questionnaire module to supplement the EORTC QLQ-C30 to assess quality of life in patients with hepatocellular carcinoma, the EORTC QLQ-HCC18. *European Journal of Cancer*.

